# Affections Likely to Occur in the Alveolar Process

**Published:** 1890-11

**Authors:** 


ARTICLE V.

### AFFECTIONS LIKELY TO OCCUR IN THE ALVEOLAR PROCESS.

Under this heading Dr. Shurer contributes a short article to the *Dental Register* for May. He considers one of the most important factors in the production of pathological conditions of the alveolar process is a diseased condition of its lining membrane—the periosteum. Speaking of necrosis, he says that there is reason to believe that the process of exfoliation is effected by the action of a corrosive fluid poured out from the fungous granulations of the living bone in contact with the necrosed part. "Necrosis of the alveolar process occurs very frequently while the system is under the influence of mercurial medicines, and during bilious and inflammatory fevers and certain other constitutional diseases, as syphilis, scurvy, smallpox, &c., and may also result from mechanical injuries. In many cases, especially of chronic disease, there is a definite thickening of the alveolar wall at or near its margin, which is clearly the result of exostosis, brought about by irritation in the immediate neighborhood. In most, if not all, of these cases, the peridental membrane will be found destroyed between this thickened rim and the root of the tooth, and if the gum be slit up and turned back, it is seen that the portion of the alveolus lying next the teeth has been absorbed. We thus have an absorption of the inner portion of the alveolar wall and at the same time a deposit of bone on the outer portion, which causes the gum tissue to be held away from the root of the tooth. This thickening of the rim is very irregular, but seldom fully encircles the teeth. And as the destructive process is going on beneath this thickening of the bone, there will often be found jagged prominences that will interfere with the healing process, unless removed. The destruction of bone in these cases seems to be a process of absorption. A tooth is occasionally slowly forced from its place by a deposit of bony matter at the sides, or more generally, at the bottom of the socket, causing a gradual displacement of the tooth. The causes are not rightly understood, though it is believed to be due to some irritation of the peridental membrane which seems to be so susceptible to morbid impressions that the pressure of a very conical root may be sufficient to produce this effect, or the pressure of a tooth possessing only a very low degree of vitality.

The writer of the paper connects this class of cases with those in which absence of all pressure causes alveolar exostosis, as, where a tooth has lost its antagonist and becomes elongated in consequence.—*London Dental Record.*

